# Luteolin Inhibits Tumorigenesis and Induces Apoptosis of Non-Small Cell Lung Cancer Cells via Regulation of MicroRNA-34a-5p

**DOI:** 10.3390/ijms19020447

**Published:** 2018-02-02

**Authors:** Ze-Qun Jiang, Mu-Han Li, Yue-Mu Qin, Hai-Ying Jiang, Xu Zhang, Mian-Hua Wu

**Affiliations:** Jiangsu Collaborative Innovation Center of Traditional Chinese Medicine (TCM) Prevention and Treatment of Tumor, Nanjing University of Chinese Medicine, 138 Xianlin Road, Nanjing 210023, China; 290343@njucm.edu.cn (Z.-Q.J.); 260261@njucm.edu.cn (M.-H.L.); qinyuemu@163.com (Y.-M.Q.); 290635@njucm.edu.cn (H.-Y.J.); 290028@njucm.edu.cn (X.Z.)

**Keywords:** luteolin, apoptosis, non-small cell lung cancer, microRNA-34a-5p

## Abstract

Luteolin (LTL) exerts remarkable tumor suppressive activity on various types of cancers, including non-small cell lung cancer (NSCLC). However, it is not completely understood whether the mechanism of its action against NSCLC is related to microRNAs (miRNAs). In the present study, we investigated the anti-tumor effects of LTL on NSCLC in vitro and in vivo. The results revealed that LTL could inhibit cell proliferation and induce apoptosis in both A549 and H460 cells. In a H460 xenograft tumor model of nude mice, LTL significantly suppressed tumor growth, inhibited cell proliferation, and induced apoptosis. miRNA microarray and quantitative PCR (qPCR) analysis indicated that miR-34a-5p was dramatically upregulated upon LTL treatment in tumor tissues. Furthermore, MDM4 was proved to be a direct target of miR-34a-5p by luciferase reporter gene assay. LTL treatment was associated with increased p53 and p21 protein expressions and decreased MDM4 protein expression in both NSCLC cells and tumor tissues. When miR-34a-5p was inhibited in vitro, the protein expressions of Bcl-2 and MDM4 were recovered, while that of p53, p21, and Bax were attenuated. Moreover, caspase-3 and caspase-9 activation induced by LHL treatment in vitro were also suppressed by miR-34a-5p inhibition. Overall, LTL could inhibit tumorigenesis and induce apoptosis of NSCLC cells by upregulation of miR-34a-5p via targeting MDM4. These findings provide novel insight into the molecular functions of LTL that suggest its potential as a therapeutic agent for human NSCLC.

## 1. Introduction

Lung cancer is one of the most common malignancies and the leading cause of cancer-related death worldwide [1]. Approximately 85% of all lung cancer cases are non-small cell lung cancer (NSCLC), which is extremely difficult to cure [2,3,4,5]. Despite great advancements in diagnostic methods, surgery, and chemotherapy and radiotherapy treatments, the 5-year overall survival rate of NSCLC patients remains frustratingly poor [6,7,8]. Therefore, there is an urgent need to discover more effective agents for NSCLC therapy.

Studies in the past decade have suggested that luteolin (3′,4′,5,7-tetrahydroxyflavone), a flavonoid which has been found in many foods and green plants, has encouraging anti-cancer effects [9]. Experimental evidence has shown that luteolin (LTL) inhibits cell proliferation or induces apoptosis in lung cancer cells by acting on several molecular targets in vitro. For instance, in human NSCLC A549 cells, LTL could obviously inhibit cell growth and activate the mitochondrial pathway of apoptosis by phosphorylating c-Jun N-terminal kinase (JNK) and inhibiting nuclear transcription factor κB (NF-κB) translocation [10]. Furthermore, it was reported that LTL played a role in inducing apoptosis and inhibiting cell migration in A549 cells via the activation of the mitogen-activated protein kinase/extracellular regulated protein kinase (MEK/ERK) signaling pathway [11]. LTL can also directly inhibit the proliferation and migration of Lewis lung carcinoma cells via the suppression of chemokine (CeC motif) ligand 2 (CCL2) expression [12]. However, although many targets have been identified, whether LTL plays a role in suppressing the NSCLC malignant behaviors in vivo is still unclear, and the underlying mechanism, particularly the involvement of microRNAs (miRNAs), is still poorly understood.

miRNAs are small non-coding RNAs that participate in various biological processes, such as proliferation, apoptosis, differentiation, and tumorigenesis [13]. They modulate gene expression at the post-transcriptional level through interacting with the 3′-untranslated regions of multiple target mRNAs [13]. Increasing studies have confirmed that miRNAs play important roles in cell survival, metastasis, epithelial–mesenchymal transition, and progression of NSCLC [14,15,16]. The miR-34 family of miRNAs consists of three members: miR-34a, miR-34b, and miR-34c. miR-34a, which functions as a potent tumor suppressor, is the predominant member of the miR-34 family [17,18,19,20]. It has been reported that miR-34a, which was decreased in NSCLC patients with metastasis, could be clinically utilized as a biomarker for the clinical prognosis or diagnosis of NSCLC [21]. This suggests that miR-34a is a potential target for the treatment of NSCLC.

Accumulating evidence has shown that LTL exerts anticancer effects via miRNA modulation in various types of cancer. It was demonstrated that LTL could inhibit the proliferation of and induce apoptosis in prostate cancer cells by downregulating the expression of miR-301 [22]. In breast cancer cells, LTL was found to decrease the expression of miR-21 to induce apoptosis [23]. In human gastric cancer cells, it was shown that LTL could upregulate miR-34a expression and downregulate Bcl-2 expression to induce apoptosis [24]. Given the potential capacity of LTL to regulate miRNA expressions, we surmised that it would be a promising agent for miRNA-based cancer therapies.

Our previous study has found that a panel of eighteen miRNAs was significantly changed after LHL treatment in H460 cells by microarray analysis (Figure S1). However, the relationship between miRNA modulation and the mechanism of action of LHL is not clear. Thus, the aim of the present study was to elucidate the in vivo and in vitro effects of LTL on NSCLC, and investigate the underlying mechanisms, particularly the involvement of miRNAs.

## 2. Results

### 2.1. LTL (Luteolin) Inhibits the Proliferation of and Induces Apoptosis in A549 and H460 Cancer Cells In Vitro

**Figure 1:**
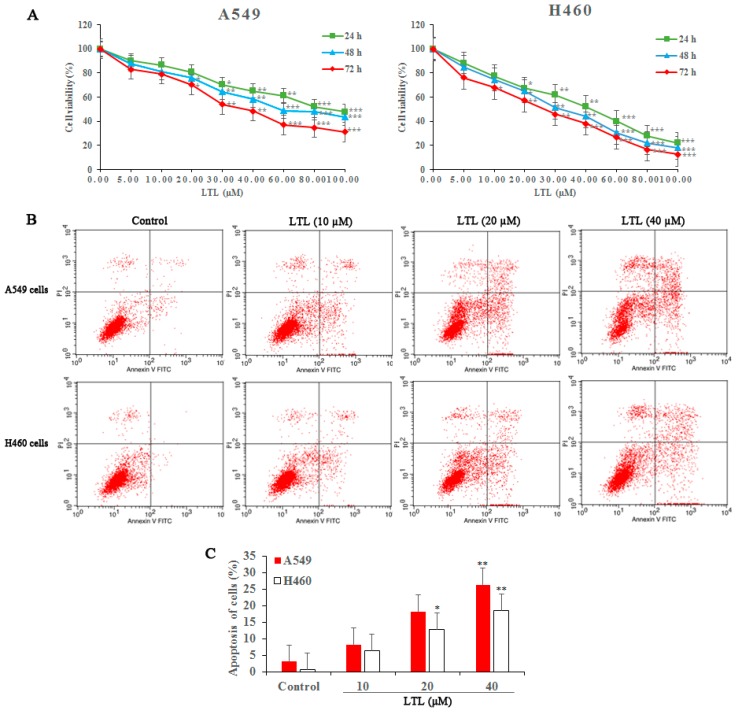
Luteolin (LTL) inhibits proliferation of and induces apoptosis in A549 and H460 cancer cells. (**A**) Cell proliferation was measured with a cell counting kit-8 (CCK-8) assay when A549 and H460 cancer cells were treated with LTL at various concentrations (5, 10, 20, 30, 40, 60, 80, and 100 μM) for up to 72 h; (**B**) Apoptosis of A549 and H460 cells was quantified 24 h after treatment with LTL at various concentrations (10, 20, and 40 µM) by Annexin V-Fluorescein (FITC)/Propidium Iodide (PI) double-staining; (**C**) Bar graphs represent the proportion of early and late stage apoptosis cells. The results are expressed as means ±Standard Deviation (SD) of three independent experiments. * *p* < 0.05, ** *p* < 0.01, *** *p* < 0.001 compared with the controls.

To investigate the role of LTL in NSCLC cell viability, cells were treated with various concentrations of LTL (0, 5, 10, 20, 30, 40, 60, 80, and 100 μM) and cell survival was measured with a cell counting kit-8 (CCK-8) assay. The results revealed a dose-dependent decrease in proliferation of A549 and H460 cells after LTL treatment (Figure 1A). As shown in Figure 1A, the half-maximal inhibitory concentration (IC_50_) for both cell lines at 48 or 72 h exposures was about 40 μM. Hence, we selected LTL from 10 to 40 µM for subsequent in vitro studies. Similarly, a progressive increase in both early and late apoptotic cell populations was observed after LTL treatment (0, 10, 20, and 40 µM) for 24 h (Figure 1B,C). 

### 2.2. LTL Inhibits Tumor Growth in the H460 Xenografts Mice Model

**Figure 2:**
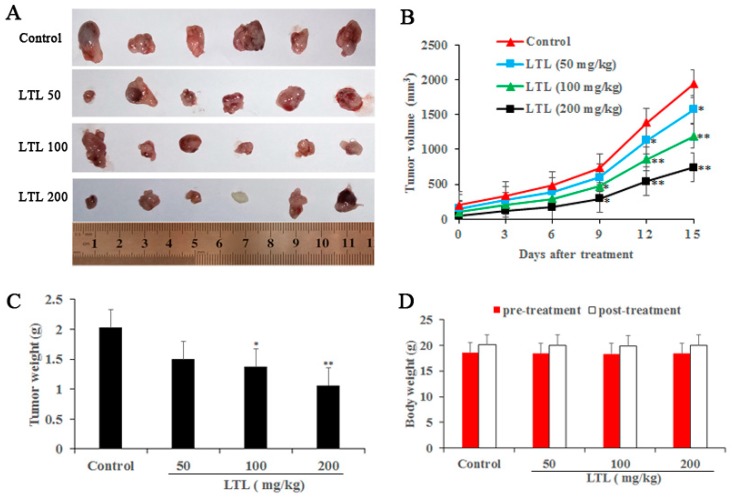
LTL inhibits tumor growth in the H460 xenografts mice model. Dissected tumors were photographed (**A**); and the tumor volume, tumor weight, and body weight from LTL-treated mice (0, 50, 100, and 200 mg/kg/day) were measured (**B**–**D**). The results are expressed as means ± SD of three independent experiments. * *p* < 0.05, ** *p* < 0.01, compared with the controls.

Next, we investigated the tumor inhibitory effect of LTL (50, 100, and 200 mg/kg/day) in vivo using the nude mice model that bore subcutaneous H460 xenografts. The anti-cancer effects of LTL were observed after 15 days of treatment, confirmed by smaller tumor volumes and lower tumor weights in the treated groups compared with the untreated control (Figure 2A–C). Moreover, the body weight of the mice had no significant changes in either the control or LTL treatment groups (Figure 2D), suggesting that this therapy was safe and well-tolerated.

### 2.3. Effect of LTL on Lung Histology

**Figure 3:**
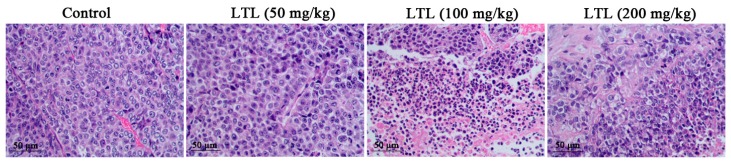
Histological analysis of tumor samples after LTL administration.

To obtain more complete information on the inhibitory effect of LTL on tumor growth, histopathological evaluation on tumor tissue sections stained with H&E was performed. As shown in Figure 3, dense viable tumor cells with a large nucleus and abundant cytoplasm were demonstrated in the control group. However, tumors treated with LTL (50, 100, or 200 mg/kg) exhibited marked inflammatory cell infiltration and more clear cell death characteristics and phenotype, especially in the LTL high-dose group (200 mg/kg).

### 2.4. LTL Treatment Promotes Apoptotic Cell Death and Inhibits Cell Proliferation

**Figure 4:**
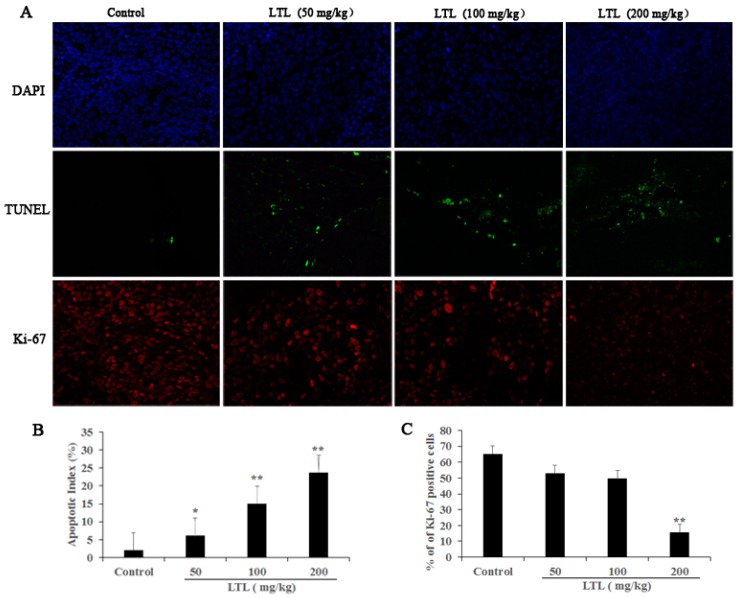
The effect of LTL on tumor cell apoptosis and proliferation in vivo. Paraffin sections of tumor tissue were tested by TUNEL (terminal deoxynucleotidyl transferase dUTP nick end labeling) and Ki-67 staining analysis. (**A**) TUNEL-positive cells (green) and Ki-67-positive cells (red) were observed under a fluorescence microscope (×400). Nuclei were counter-stained with DAPI (blue); (**B**) The apoptotic index was calculated as the number of TUNEL-positive cells for each group; (**C**) Quantification of Ki-67-positive cells is represented as the ratio of Ki-67-positive cells to the total number of cells for each group. The results are expressed as means ± SD of three independent experiments. * *p* < 0.05, ** *p* < 0.01, compared with the controls.

To determine the mechanisms of the anti-cancer effect of LTL treatment, we examined its effects on tumor cell apoptosis and proliferation. As shown in Figure 4A,B, immunofluorescence images of TUNEL (Roche, Manheim, Germany) staining revealed a visible increase of green fluorescence signals in tumor tissues of the LTL groups compared to the control group, which was indicative of apoptosis. Meanwhile, treatment with different doses of LTL resulted in an apparent decrease of red fluorescence signals in LTL-treated tumor tissues compared to the control group using Ki-67 staining (Figure 4A). Quantification revealed that LTL treatment reduced proliferation of lung cancer cells in a dose-dependent manner (Figure 4C). These results indicated that LTL exerts pro-apoptotic and anti-proliferation effects in vivo.

### 2.5. Expression of MiRNAs Changes in Response to LTL in H460 Tumor Xenografts

**Figure 5:**
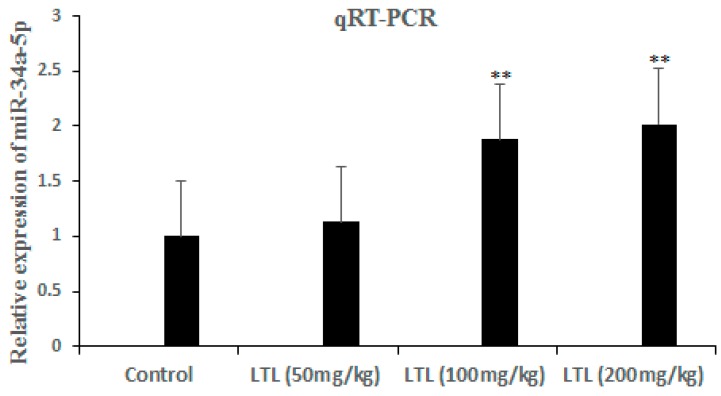
The levels of miR-34a-5p were detected by qRT-PCR in H460 tumor Xenografts. The fold change for miR-34a-5p in LTL-treated groups relative to the vehicle control was analyzed using the 2^−ΔΔ*C*t^ method. The results are expressed as means ± SD of three independent experiments. ** *p* < 0.01, compared with the controls.

Our previous study has indicated that LTL upregulated miR-34a-5p and other miRNAs expression in H460 cells by microarray analysis (Figure S1). By microarray analysis of H460 tumor xenografts, we revealed that compared with the control group, 20 miRNAs, including miR-34a-5p, were significantly upregulated and 4 miRNAs were significantly downregulated by the LTL high-dose group (200 mg/kg) (Table 1). Although miR-34a-5p is not the most responsive miRNA, it is the only miRNA which is consistent with our in vitro microarray study (Figure S1). To verify that LTL treatment upregulated miR-34a-5p, the levels of miR-34a-5p were measured by quantitative real-time PCR. As shown in Figure 5, the expression of miR-34a-5p was confirmed to be upregulated in a dose-dependent manner after LTL treatment in vivo.

### 2.6. MDM4 Is a Direct Downstream Target of miR-34a-5p

**Figure 6:**
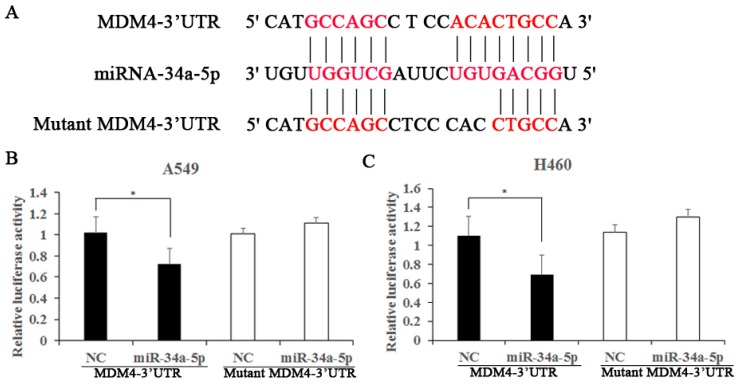
MDM4 is a direct target of miR-34a-5p in A549 and H460 cells. (**A**) The sequence of miR-34a-5p and its binding sites in the 3′-UTR of MDM4 mRNA; The red letters stand for complementary base pairing between miRNA-34a-5p and 3′-UTR of MDM4 mRNA; (**B**,**C**) Luciferase activity was measured in A549 and H460 cells with a dual-luciferase reporter assay. The MDM4-3′-UTR or the mutant MDM4-3′-UTR vectors and the miR-34a-5p mimics or negative controls were co-transfected into cells with Lipofectamine 2000. The relative luciferase activity was normalized to the Renilla luciferase activity. The results are expressed as means ± SD of three independent experiments. * *p* < 0.05, compared with the controls.

To identify the molecular mechanism responsible for the biological functions of miR-34a-5p in NSCLC, we next explored the target genes of miR-34a-5p by using public databases, including miRBase (http://www.mirbase.org/) and TargetScan (http://www.targetscan.org/). The bioinformatics analysis showed that MDM4 contained a complementary binding site for miR-34a-5p in its 3′-UTR (Figure 6A). To confirm whether MDM4 is a target of miR-34a-5p, dual-luciferase reporter assays were performed in A549 and H460 cells. The results demonstrated that co-transfection of miR-34a-5p significantly reduced MDM4 3′-UTR luciferase activity in both cell lines compared with the negative control (Figure 6B,C). However, there was no obvious difference on the luciferase activity of mutated MDM4 3′-UTR in which the miR-34a-5p binding site was disrupted (Figure 6B,C). These findings suggest that MDM4 is a direct downstream target of miR-34a-5p.

### 2.7. LTL Increases the Expression of P53 and P21 and Decreases the Expression of MDM4 In Vivo

**Figure 7:**
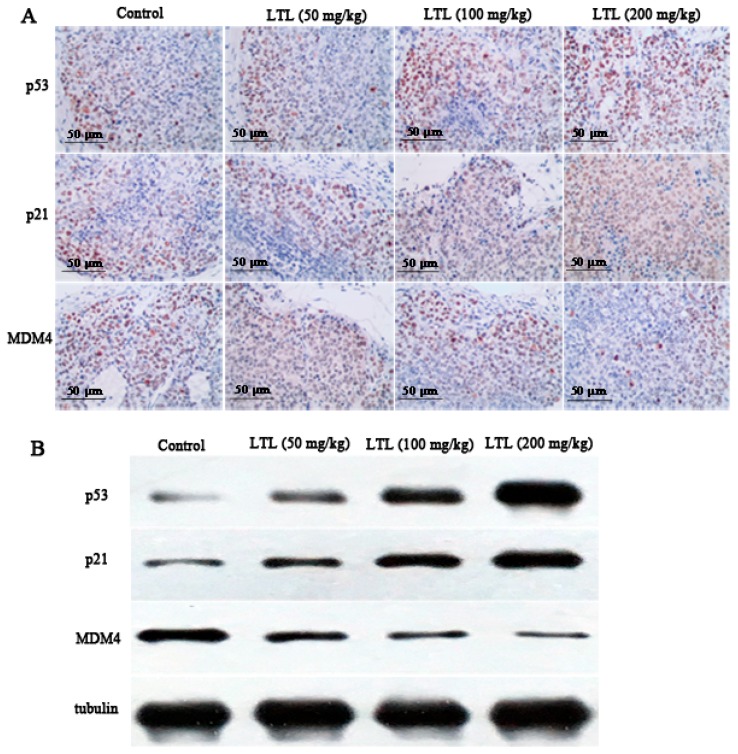
Effect of LTL on the expression of p53, p21, and MDM4 in Vivo. Tumor tissue sections were tested by immunohistochemistry and Western blot analysis, respectively. (**A**) Representative photographs of the tumor sections from LTL-treated groups examined by immunohistochemical staining; (**B**) Tissue lysates were separated by SDS-PAGE and analyzed on Western blots with the indicated antibodies. Tubulin was used as a loading control.

The above-mentioned results indicated that the miR-34a-5p-MDM4 pathway might be a potential therapeutic target for NSCLC. To further address the treatment mechanism and the downstream targets of this pathway, immunohistochemistry and a Western blot analysis were performed to detect the expression of p53, p21, and MDM4 in the xenograft specimens. Data from the immunohistochemical assay revealed that there were substantially more p53- and p21-positive cells in tumor tissue sections from LTL-treated groups (Figure 7A). Meanwhile, the expression of MDM4, a target of miR-34a-5p, was decreased after different doses of LTL treatment in H460 xenograft tissues (Figure 7A). Similar results were observed in the Western blot analysis (Figure 7B). These data suggest that p53, p21, and MDM4 may participate in LTL-induced apoptosis in vivo.

### 2.8. miR-34a-5p Participates in LTL-Induced Apoptotic Effects by Regulating Its Downstream Target Protein Expressions In Vitro

**Figure 8:**
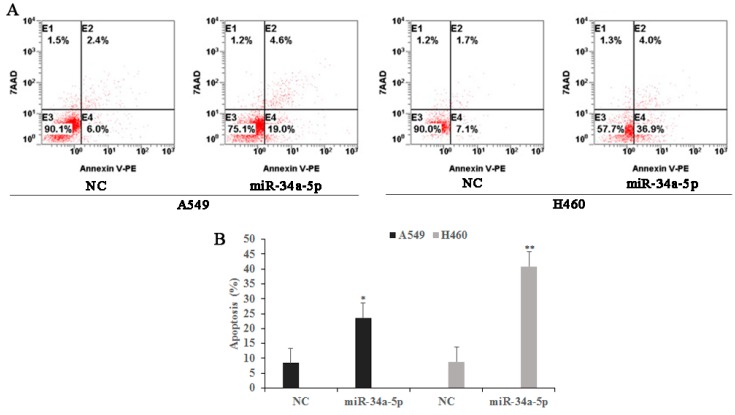
The effects of miR-34a-5p on tumor cell apoptosis in A549 and H460 cells. Both cells were transfected with either a miR-34a-5p expression vector or a miR-34a-5p negative control. (**A**) Twenty-four hours after transfection, cell apoptosis was measured by flow cytometry; (**B**) Bar graphs represent the proportion of early and late stage apoptosis cells. The results are expressed as means ± SD of three independent experiments. * *p* < 0.05, ** *p* < 0.01, compared with the negative controls (NCs).

To further explore the biological role of miR-34a-5p in NSCLC, A549 and H460 cells were transfected with either a miR-34a-5p expression vector or a miR-34a-5p negative control. As measured by flow cytometry, miR-34a-5p overexpression increased early and late apoptotic rates in both cell lines (23.6% and 40.9% apoptosis, respectively, compared with 8.4% and 8.8% apoptosis for negative control (NC) cells, respectively) (Figure 8).

**Figure 9:**
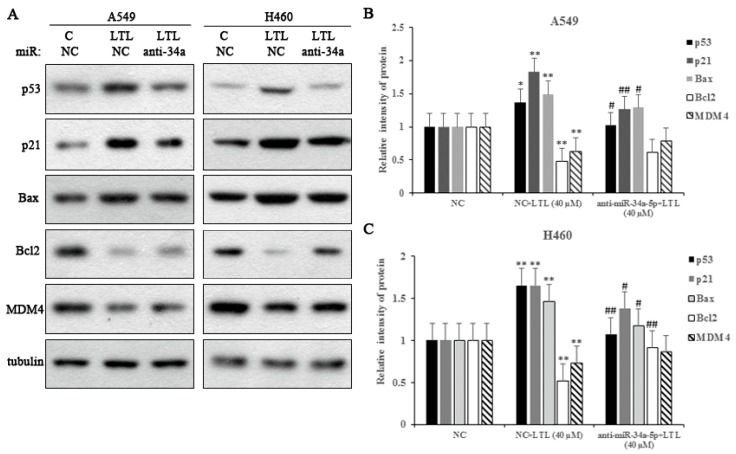
Effect of miR-34a-5p inhibition on apoptosis-related protein expressions in vitro. (**A**) Protein expression levels of p53, p21, Bax, Bcl-2, and MDM4 in A549 and H460 cells treated with anti-34a (LV-hsa-miR-34a-5p-inhibition) or NC (negative control) after LTL (40 μM) treatment for 24 h. Tubulin was used as an internal control; (**B**,**C**) Histograms represent quantification of the relative expression of the proteins. The results are expressed as means ± SD of three independent experiments. * *p* < 0.05, ** *p* < 0.01, compared with NC group; ^#^ *p* < 0.05, ^##^ *p* < 0.01, compared with NC + LTL (40 μM) group. C: control.

Next, we transfected miR-34a-5p inhibitor into NSCLC A549 and NCI-H460 cells to test the effects of miR-34a-5p on apoptosis-related protein expressions. As shown in Figure 9, LTL treatment (40 µM) significantly increased the expression of p53, p21, and Bax proteins in both cell lines. Moreover, the expression of Bcl-2 and MDM4 proteins were downregulated compared with the control. However, LV-hsa-miR-34a-5p-inhibition (anti-34a) completely reversed these effects. The results indicated that miR-34a-5p inhibition decreased p53, p21, and Bax expression and attenuated reductions in Bcl-2 and MDM4 protein levels (Figure 9). Taken together, the data indicate that miR-34a-5p participated in LTL-induced apoptotic effects by regulating its downstream targets.

### 2.9. miR-34a-5p Participates in LTL-Induced Apoptotic Effects through Regulation of Caspase-3 and Caspase-9 Activities

**Figure 10:**
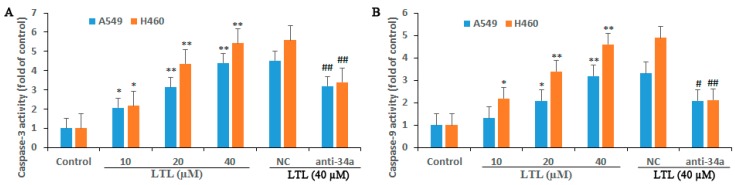
Effect of miR-34a-5p inhibition on apoptosis-related caspase activation in vitro. Caspase-3 (**A**) and Caspase-9 (**B**) activities were determined using colorimetric assay kits as described in the method section for A549 and H460 cell lines treated with anti-34a (LV-hsa-miR-34a-5p-inhibition) or NC (negative control) after LTL treatment (10, 20, or 40 μM) for 48 h. The results are expressed as means ± SD of three independent experiments. * *p* < 0.05, ** *p* < 0.01, compared with control group; ^#^ *p* < 0.05, ^##^ *p* < 0.01, compared with NC + LTL (40 μM) group.

Cysteine aspartases (caspases) are known to be the executioners of apoptosis. The role of caspase-3 and caspase-9 in LTL-induced apoptosis was confirmed using LV-hsa-miR-34a-5p-inhibition. As shown in Figure 10, caspase-3 and caspase-9 activities were dose-dependently increased in A549 and H460 cells after incubation with LHL (10, 20, and 40 μM) for 48 h. However, the caspases activation induced by LHL (40 μM) were reduced significantly when LV-hsa-miR-34a-5p-inhibition (anti-34a) was applied (Figure 10). The results suggested that the elevation of caspase-3 and caspase-9 activities was induced by LHL and that LV-hsa-miR-34a-5p-inhibition had a suppressive effect on LHL-related activation of caspases.

## 3. Discussion

Currently, the prognosis or therapy for patients with advanced stage NSCLC is poor. In recent years, attempts to improve survival rates in NSCLC have focused on innovative therapeutic methods. Compounds of natural origin have proven to be an attractive option for cancer therapy due to their tumor suppressive activity, safety, and inexpensiveness. One such natural agent is LTL, which has attracted much attention for its anti-cancer effects in many malignancies [9,12,22,23,24]. Herein, our results showed that LTL inhibited tumor growth and exhibited remarkable apoptotic activity against NSCLC in vivo and in vitro.

As expected, LTL administration significantly reduced tumor growth as evidenced by a decrease in tumor volume and tumor weight in H460 cell xenografts. Consistently, the H&E analysis revealed that LTL increased inflammatory cell infiltration and apoptotic death in xenografted tumor tissues. The TUNEL assay results further confirmed that LTL could promote apoptosis of tumor cells in vivo. Moreover, the immunofluorescence staining with Ki-67 revealed a visible decrease in tumor cell proliferation, confirming the anti-proliferative activity of LTL in NSCLC. By microarray and qRT-PCR assays, we reported for the first time that the expression of miR-34a-5p was significantly increased after LTL treatment in vivo, which was consistent with our previous findings in vitro (Figure S1).

Growing evidence suggests that miR-34a is a potent tumor suppressor molecule [17,18,19,20]. It has been proven that the ectopic expression of miR-34a affects cell viability, proliferation, and migration in human esophageal cancer [25]. Another report indicated that the increased expression of miR-34a could induce cell apoptosis and inhibit cell proliferation and invasion in Hep2 laryngeal carcinoma cells [26]. The underlying mechanisms that account for the miR-34a-induced tumor malignancy suppression have not been fully identified, but increasing studies have demonstrated that miR-34a is under the direct control of tumor suppressor p53 [18,19,20,21]. It has been reported that miR-34a-5p could suppress colorectal cancer metastasis by inhibiting cell proliferation, migration, and invasion in a p53-dependent manner [27]. The miRNA target site of miR-34a-5p was identified within the 3′-UTRs of p53-induced mRNAs, such as Bcl2 [28]. MiR-34a-5p overexpression was confirmed to suppress ovarian cancer (OC) cell growth and induce apoptosis by directly targeting Bcl2 [29]. Herein, our findings showed that overexpression of miR-34a-5p promoted apoptosis of both A549 and H460 cells in vitro. By performing dual-luciferase reporter gene assays, we demonstrated that MDM4 is a direct downstream target for miR-34a-5p. Western blot analysis and immune staining of the tumor sections revealed a decrease of MDM4 and an increase of p53 and p21 after LTL treatment. It is well-known that p53, a tumor suppressor gene, plays a pivotal role in the regulation of cell growth arrest, apoptosis, and DNA damage repair. The primary function of p53 is known to activate the transcription of genes that can promote cell-cycle arrest, apoptosis, and the repression of tumor progression [30]. For example, the cyclin-dependent kinase inhibitor p21 (WAF1/CIP1) is a direct transcription target of p53. Upon activation of the p53 pathway, an upregulation of p21 protein is induced, which leads to cell-cycle arrest or apoptotic cell death [31]. In contrast, MDM4, a potential oncogene, is known to repress p53 gene transcription and promote its proteasomal degradation [32]. It is thus likely that MDM4-targeted agents that can inhibit MDM4 expression, thereby reactivating p53, could lead to cancer cell death. Therefore, our results indicate that LTL could inhibit cell proliferation and promote apoptosis by the modulation of miR-34a-5p and its downstream targets in NSCLC.

Recent studies have demonstrated that p53 and miR-34a may act cooperatively to inhibit cell proliferation through regulating multiple transcriptional targets. Chakraborty et al. found that the p53-miR-34a-Bcl2 regulatory axis might be critical in sensitizing drug-resistant NSCLC cells to capsaicin [33]. Research by Hu et al. demonstrated that 5-Aminolevulinic acid (ALA)-Sonodynamic therapy (SDT) induced apoptotic cell death and inhibited proliferation via the p53-miR-34a-Sirt1 axis in malignant melanoma [34]. These effects may be explained by the regulation of apoptosis- and cell-cycle-related miR-34a targets, such as Bcl2, Sirt1, and MDM4. In the current study, we found that the expression levels of miR-34a-5p targets, such as Bcl2 and MDM4, were decreased, while the expressions of p53, p21, and Bax were increased after LTL treatment in A549 and H460 cells. This is consistent with our findings in vivo.

Emerging evidence indicates that there is a functional link between p53, miR-34a, and MDM4: miR-34a is a p53 target and suppresses MDM4 expression, whereas MDM4 inhibits p53 transcriptional activity [35]. Loss of MDM4 could induce p53 acetylation, resulting in activation of the p53 pathway [35]. It has been previously demonstrated that activated p53 induces the expression of miR-34a, and then miR-34a contributes to the pro-apoptotic effect of p53 [19,20]. The downstream effectors of p53 include p21, the pro-apoptotic protein Bax, and the anti-apoptotic protein Bcl2. The downregulation of Bcl2 and upregulation of p21 and Bax all contribute to p53-mediated apoptosis [36]. This is consistent with our above findings. In our experimental results, we also observed that when anti-miR-34a-5p was added, the Bcl2 and MDM4 protein expressions were recovered, but the expressions of p53, p21, and Bax were attenuated in A549 and H460 cells. It is therefore tempting to speculate that miR-34a-5p potentially plays an important role in the regulation of apoptosis-related gene expressions in NSCLC.

Despite varying conditions under which apoptosis can occur, the activation of caspase family members is a critical determinant. Caspase-3 and -9 are situated at key junctions in apoptotic pathways. Caspase-3 is an indispensable effector of programmed cell death, which can be directly cleaved and activated by the initiator caspase-9 [37]. In the current study, the activation of caspase-3 and caspase-9 were confirmed after LTL treatment in both A549 and H460 cells. Addition of the miR-34a-5p inhibitor significantly antagonized the caspases activation induced by LTL, indicating that miR-34a-5p may participate in LHL-induced apoptosis by the activation of caspase-9 and -3. Evidence is emerging that mitochondria play a key role in the activation of caspase-9 and the subsequent activation of effector caspase-3, leading to downstream apoptotic responses. The mitochondrial signaling pathway has been shown to be regulated by key proteins associated with apoptosis, such as Bax, Bcl-2, and cytochrome c, with subsequent activation of caspase-3 and poly (ADP) ribose polymerase (PARP) [38]. It has been reported that LTL induced apoptosis in A549 cells by activating caspase-9 through a mitochondria-dependent (intrinsic) death pathway [39]. A concomitant increase in the levels of caspase-3 and cleaved PARP were also observed [39]. Our data suggest that altering the expression of Bax, Bcl-2, and the activation of caspase-3 might be at least contributing part of the mechanism underlying the apoptotic activity of LHL. It is thus tempting to speculate that miR-34-5p may be associated with the mitochondrial pathway in NSCLC. However, the role of miR-34a-5p in modulating other proteins related to the mitochondrial apoptotic pathway remains to be confirmed in our future studies.

## 4. Materials and Methods

### 4.1. Reagents and Antibodies

Dimethyl sulfoxide (DMSO), trypsin, penicillin, and streptomycin were obtained from Sigma-Aldrich (St. Louis, MO, USA). The Roswell Park Memorial Institute (RPMI) 1640 medium and fetal bovine serum (FBS) were purchased from Hyclone (Logan, UT, USA). The Cell Counting Kit-8 (CCK-8) was from KeyGen Biotech (Nanjing, China). The Annexin V-PE/7-AAD apoptosis detection kit was obtained from YEASEN (Shanghai, China). Antibodies against p53, p21, MDM4, Bax, and Bcl-2 were all purchased from Cell Signaling Technology (Danvers, MA, USA). Antibody against tubulin was from Sigma. Anti-Ki-67 antibody was purchased from Abcam (ab16667). All of the secondary antibodies were obtained from Santa Cruz Biotechnology (Dallas, TX, USA). Diaminobenzidine (DAB) reagent was from Long Island Biotec (Shanghai, China). The DeadEnd™ Fluorometric TUNEL System was from Roche (Manheim, Germany). Trizol was from Invitrogen (Carlsbad, CA, USA). The miRNA ULSTM Labeling Kit was purchased from Kreatech Diagnostics (Amsterdam, The Netherlands). Mouse miRNA OneArray^®^ v5 was obtained from Phalanx Biotech Group (Hsinchu, Taiwan). A RevertAid First Strand cDNA Synthesis Kit and Lipofectamine 2000 were purchased from Thermo Fisher Scientific (Waltham, MA, USA). The SYBR green qPCR master mix kit was from Fermentas (Burlington, MD, USA). The pGL3 control vector and the dual-luciferase reporter assay system were from Promega (Madison, WI, USA). The enhanced chemiluminescence system was obtained from Pierce (Rockford, IL, USA). The caspase-3 and caspase-9 assay kits were from KeyGen Biotech.

Sodium carboxymethyl cellulose (CMC) and LTL (>98% pure) were purchased from Aladdin Industrial Corporation (Shanghai, China). For cell culture use, LTL was dissolved in DMSO to make a 1 M stock solution, aliquoted, and stored at −20 °C.

### 4.2. Cell Culture

Human NSCLC A549 and H460 cells were purchased from the American Type Culture Collection (ATCC). Cells were routinely cultured in RPMI-1640 medium supplemented with 10% FBS, 100 U/mL penicillin, and 100 μg/mL streptomycin in a humidified 37 °C incubator with 5% CO_2_.

### 4.3. Cell Viability Assay

The cell viability was determined using a CCK-8 assay as described previously [40]. Briefly, cells were seeded in 96-well plates at a density of 8000 cells per well. Samples of subconfluent cells were treated with various concentrations of LTL (0, 5, 10, 20, 30, 40, 60, 80, and 100 µM) for 24, 48, and 72 h, respectively. Before terminating the cell culture, CCK-8 assay reagent (10 μL) was added to each well. The optical density (OD) values were determined at 450 nm and the relative cell viability was assessed by the following equation: Cell viability (%) = (OD_LTL groups_/OD_control groups_) × 100.

### 4.4. Apoptosis Assay

Cells (1.5 × 10^5^ cells per well) were seeded in 6-well plates and treated with different concentrations of LTL (0, 10, 20, and 40 µM) for 24 h. Subsequently, cells were harvested, and apoptosis was quantitatively estimated with the Annexin V-PE/7-AAD apoptosis detection kit according to the manufacturer’s instructions. The analysis of apoptotic cells was performed using FASS-Calibur flow cytometry (BD Biosciences, San Jose, CA, USA).

### 4.5. Tumor Xenograft Model in Mice

Forty male BALB/c nude mice aged 3 to 5 weeks were purchased from Cavens Laboratory Animal Co., Ltd. (Changzhou, China) and housed in individually ventilated cages. To create the tumor model, H460 tumor cells (2 × 10^6^ cells in 100 mL of serum-free culture medium) were subcutaneously injected into the right-side flanks of the mice. Two weeks after injection, the animals were randomly allocated to the control and three experimental groups (*n* = 10 mice/group), which received different concentrations of LTL (50, 100, and 200 mg/kg/day, respectively). The mice were treated daily with intragastrically administered 0.5% CMC or LTL dissolved in 0.5% CMC for the next fifteen days. The volume of the tumor was monitored every 3 days and calculated with the following formula: volume = (length × width^2^)/2. At the end of study period, the mice were sacrificed by cervical vertebral dislocation. The tumors were extracted, weighed, and frozen in liquid nitrogen or fixed in 10% buffered neutral formalin for further analysis. All animal procedures and experimental protocols were approved by the Laboratory Animal Ethics Committee of Nanjing University of Chinese medicine (ACU170602; approved on 26 June 2017). 

### 4.6. Histological Evaluation, Immunohistochemistry, and Western Blot Analysis

Tumor tissues fixed in 10% formalin were embedded in paraffin and cut into 4-µm-thick sections. For histological examination, the sections were stained by a routine hematoxylin and eosin (H&E) procedure, and then examined under a light microscope.

For the immunohistochemistry (IHC) assay, paraffin sections of tumor samples were dewaxed in xylene and rehydrated through graded ethanol. Antigens were retrieved in sodium citrate buffer for 20 min in a microwave, and then the endogenous peroxidase activity was blocked by incubation with 3% hydrogen peroxide for 10 min. Next, sections were blocked with normal goat serum for 30 min at room temperature and then incubated with the specific primary antibodies at 4 °C overnight. After three washes in PBS for 3 min, sections were incubated with HRP-conjugated secondary antibodies at 37 °C for 60 min. DAB reagent was used to visualize the signal followed by counterstaining with hematoxylin.

Tissue lysates of each group were prepared in lysis buffer and Western blot analyses were performed as described previously [40]. After blocking with 5% skim milk solution, the membranes were blotted respectively with different primary antibodies overnight at 4 °C and incubated subsequently with the corresponding HRP-labeled anti-mouse or anti-rabbit secondary antibodies for one hour at room temperature. The protein bands were visualized by an enhanced chemiluminescence system and densitometry was quantified with Image J software (NIH, Bethesda, MD, USA, 1.33u). The protein levels were normalized to that of tubulin. 

### 4.7. In Situ Terminal Deoxynucleotidyl Transferase dUTP Nick End Labeling (TUNEL) Assay and Evaluation of Ki-67 Proliferation

Nuclear DNA fragmentation in tumor tissues was detected using a DeadEnd™ Fluorometric TUNEL System according to the manufacturer’s instructions. Briefly, paraffin-embedded sections were deparaffinized and hydrated in a series of graded ethanols and then digested with trypsin for 40 min at room temperature. The tissue sections were then incubated with TUNEL reaction buffer in a 37 °C humidified atmosphere for 60 min, washed with PBS, and then incubated with DAPI for 1 min at room temperature. Tumor proliferation was identified by anti-Ki-67 antibody staining according to the manufacturer’s protocol. Slides were visualized under a fluorescence microscope (OLYMPUS CX41, Tokyo, Japan). TUNEL-positive or Ki-67-positive cells were counted at ×400 magnification. The apoptotic index or proliferation index was then defined as a ratio of (the number of apoptotic TUNEL-positive or Ki-67-positive cells)/(the total number of cells) in each field.

### 4.8. Microarray Analysis

Total RNAs of tissue samples from two groups (Control, LTL (200 mg/kg) group) with three samples per group were isolated using Trizol according to the manufacturer’s protocol. Small RNA fragments were enriched by NanoSep 100K (Pall Corporation, New York, NY, USA) and de-salted by flowing through vivaspin 500 3k (Sartorius Stedim Biotech, Goettingen, Germany) from 2.5 μg total RNA. Fluorescent targets were prepared using a miRNA ULSTM Labeling Kit. Labeled Fluorescent targets were hybridized to pre-hybridized Mouse miRNA OneArray^®^ v5. After 16 h hybridization at 37 °C, non-specific binding targets were washed away, and the slides were dried by centrifugation and scanned by an Axon 4000B scanner (Molecular Devices, Sunnyvale, CA, USA). The Cy5 fluorescent intensities of each spot were analyzed by GenePix 4.1 software (Molecular Devices), which performed median normalization.

### 4.9. Quantitative Real-Time PCR

Total miRNAs were extracted from tumor tissues with the TRIzol reagent according to the manufacturer’s instructions. First-strand cDNA was prepared from 1 μg of total RNA with a RevertAid First Strand cDNA Synthesis Kit (Thermo Fisher Scientific). Real-time PCR was carried out using a SYBR green qPCR master mix kit. The primers used were listed as follows: miR-34a-5p forward: 5′-ACACTCCAGCTGGGCAATCAGCAAGTATAC-3′; reverse: 5′-CTCAACTGGTGTCGTGGAGTCGGCAATTCAGTTGAGAGGGC-3′; U6 forward: 5′-CTCGCTTCGGCAGCACA-3′; reverse: 5′-AACGCTTCACGAATTTGCGT-3′. The relative miR-34a-5p level was normalized to U6 expression. The fold change for miR-34a-5p in LTL-treated groups relative to the vehicle control was analyzed using the 2^−ΔΔ*C*t^ method.

### 4.10. Luciferase Reporter Gene Assay

The wild-type (wt) or the mutated type (mt) of MDM4 3′-UTR containing miR-34a-5p binding sites was synthesized and inserted into the pGL3 control vector. Then, A549 and H460 cells (1 × 10^5^) were seeded into 24-well plates, respectively. The wt MDM4-3′-UTR or the mt MDM4-3′-UTR vectors (500 ng) and the miR-34a-5p mimics or negative controls were co-transfected into cells with Lipofectamine 2000. After 48 h of incubation, the cells were harvested and the luciferase activity was detected with the dual-luciferase reporter assay system. Firefly luciferase activity was normalized to the Renilla luciferase activity for each transfected well.

### 4.11. Microrna Inhibition, Transfection, and Western Blot Analysis In Vitro

Synthetic LV-hsa-miR-34a-5p-inhibition was constructed in GeneChem (Shanghai, China). The A549 and H460 cells were grown to 60% confluence in 6-well plates. The 20 nM LV-hsa-miR-34a-5p-inhibition (anti-34a) or negative control (NC) was transfected with enhanced infection solution (GeneChem) following the manufacturers’ protocol. The transfected cells in the LTL group were incubated with 40 µM LTL for 24 h, and then a Western blot analysis was used to characterize protein expressions.

A549 and H460 cell lysates of each group were prepared in lysis buffer and Western blot analyses were performed as described previously [40]. After blocking with 5% skim milk solution, the membranes were blotted respectively with different primary antibodies overnight at 4 °C and incubated subsequently with corresponding HRP-labeled anti-mouse or anti-rabbit secondary antibodies for one hour at room temperature. The protein bands were visualized by an enhanced chemiluminescence system and densitometry was quantified with Image J software (NIH, 1.33u). The protein levels were normalized to that of tubulin.

### 4.12. Caspase-3 and -9 Assay

Caspase activity was measured by using Caspase-3 and -9 assay kits according to the manufacturer’s instructions. Briefly, after the respective treatment, cells were collected and washed twice with PBS. The cells were cracked in the ice bath for 60 min. After centrifugation at 10,000 rpm for 1 min, the supernatant was used for the determination of caspase-3 or caspase-9 activity. The activity of caspase was measured at a wavelength of 405 nm using an enzyme-immunoassay instrument (TECAN, Mannedorf, Switzerland).

### 4.13. Statistical Analysis

Each experiment was performed at least three times independently. Data were presented as mean ± standard deviation and analyzed with GraphPad Prism 5 software (San Diego, CA, USA). One-way analysis of variance (ANOVA) and Student’s *t*-test were used to determine the levels of statistical differences. A value of *p* < 0.05 was considered statistically significant.

## 5. Conclusions

Taken together, our data demonstrate that LTL inhibits tumor growth by inhibiting NSCLC cell proliferation and promoting apoptosis in vivo and in vitro. We propose that miR-34a-5p plays a key role in LTL-induced antitumor effects via targeting MDM4 and enhancing the activation of the p53 pathway. Thus, our investigation provides new insights into the mechanism of LTL against NSCLC, and supports miR-34a-5p as a therapeutic target in NSCLC.

## Figures and Tables

**Table 1:** Significantly upregulated or downregulated miRNAs upon LTL (luteolin) treatment (200 mg/kg) in vivo. Arrows represent upregulation (↑), or downregulation (↓).

Name of miRNA	Fold Change	*p* Value
miR-100-5p	↑2.92	0.0135
miR-668-3p	↑3.98	0.0119
let-7i-5p	↑5.169	0.0199
let-7b-5p	↑4.80	0.0146
let-7a-5p	↑3.977	0.0086
let-7f-5p	↑5.443	0.0203
miR-451a	↑5.635	0.0336
miR-34a-5p	↑2.187	0.004
miR-222-3p	↑3.995	0.0054
miR-181a-5p	↑5.376	0.015
miR-92a-3p	↑6.915	0.0177
miR-93-5p	↑4.142	0.0016
miR-20a-5p	↑5.275	0.0019
miR-17-5p	↑5.544	0.001
miR-106a-5p	↑4.60	0.0148
miR-19b-3p	↑5.216	0.0085
miR-1983	↑4.929	0.0039
miR-208a-5p	↑4.872	0.0291
miR-7026-5p	↑6.357	0.0052
miR-6241	↑4.03	0.007
miR-3470a	↓6.97	0.0367
miR-3470b	↓5.459	0.0089
miR-3472	↓4.82	0.0061
miR-3105-5p	↓3.75	0.0021

## Supplementary Materials

Supplementary materials can be found at http://www.mdpi.com/1422-0067/19/2/447/s1.

## Abbreviations

LTL	Luteolin
NSCLC	Non-small cell lung cancer
qPCR	Quantitative PCR
JNK	c-Jun N-terminal kinase
NF-κB	Nuclear transcription factor κB
MEK/ERK	Mitogen-activated protein kinase/extracellular regulated protein kinase
CCL2	Chemokine (CeC motif) ligand 2
OC	Ovarian cancer
TUNEL	Terminal deoxynucleotidyl transferase dUTP nick end labeling
DMSO	Dimethyl sulfoxide
RPMI	Roswell Park Memorial Institute
FBS	Fetal bovine serum
DAB	Diaminobenzidine
CMC	Sodium carboxymethyl cellulose
CCK-8	Cell counter kit-8
ATCC	American type culture collection
IHC	Immunohistochemistry
NC	Negative control
PARP	Poly (ADP) ribose polymerase
