# Supplementary material for: Luteolin Inhibits Tumorigenesis and Induces Apoptosis of Non-Small Cell Lung Cancer Cells via Regulation of MicroRNA-34a-5p
Source: Int J Mol Sci. 2018 Feb 2;19(2):447. doi: 10.3390/ijms19020447 (PMC5855669; doi:10.3390/ijms19020447)
Supplement: Supplementary file 1 [file ijms-19-00447-s001.pdf]

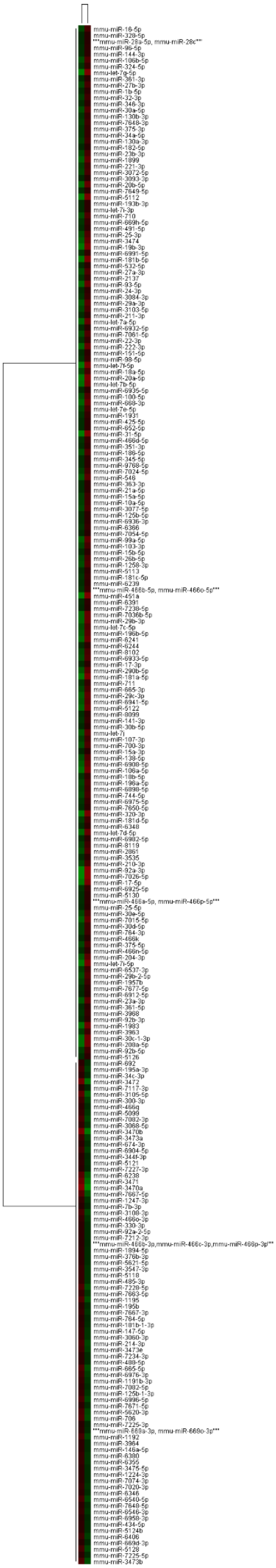
mmu-miR-16-5p
mmu-miR-328-5p
mmu-miR-28a-5p, mmu-miR-28c
mmu-miR-96-5p
mmu-miR-144-3p
mmu-miR-106b-5p
mmu-miR-324-5p
mmu-let-7g-5p
mmu-miR-361-3p
mmu-miR-27b-3p
mmu-miR-1b-5p
mmu-miR-32-3p
mmu-miR-346-3p
mmu-miR-30a-5p
mmu-miR-130b-3p
mmu-miR-7648-3p
mmu-miR-375-3p
mmu-miR-34a-5p
mmu-miR-130a-3p
mmu-miR-182-5p
mmu-miR-23b-3p
mmu-miR-1899
mmu-miR-221-3p
mmu-miR-3072-5p
mmu-miR-3093-3p
mmu-miR-20b-5p
mmu-miR-7649-5p
mmu-miR-5112
mmu-miR-193b-3p
mmu-let-7i-3p
mmu-miR-710
mmu-miR-669h-5p
mmu-miR-491-5p
mmu-miR-25-3p
mmu-miR-3474
mmu-miR-19b-3p
mmu-miR-6991-5p
mmu-miR-181b-5p
mmu-miR-532-5p
mmu-miR-27a-3p
mmu-miR-2137
mmu-miR-93-5p
mmu-miR-24-3p
mmu-miR-3084-3p
mmu-miR-29a-3p
mmu-miR-3103-5p
mmu-miR-211-3p
mmu-let-7a-5p
mmu-miR-6932-5p
mmu-miR-7061-5p
mmu-miR-22-3p
mmu-miR-222-3p
mmu-miR-151-5p
mmu-miR-98-5p
mmu-let-7f-5p
mmu-miR-18a-5p
mmu-miR-20a-5p
mmu-let-7b-5p
mmu-miR-6935-5p
mmu-miR-100-5p
mmu-miR-668-3p
mmu-let-7e-5p
mmu-miR-1931
mmu-miR-425-5p
mmu-miR-652-5p
mmu-miR-31-5p
mmu-miR-466d-5p
mmu-miR-351-3p
mmu-miR-186-5p
mmu-miR-345-5p
mmu-miR-9768-5p
mmu-miR-7024-5p
mmu-miR-546
mmu-miR-363-3p
mmu-miR-21a-5p
mmu-miR-15a-5p
mmu-miR-10a-5p
mmu-miR-3077-5p
mmu-miR-125b-5p
mmu-miR-6936-3p
mmu-miR-6366
mmu-miR-7054-5p
mmu-miR-99a-5p
mmu-miR-103-3p
mmu-miR-15b-5p
mmu-miR-26b-5p
mmu-miR-1258-3p
mmu-miR-5113
mmu-miR-181c-5p
mmu-miR-6239
mmu-miR-466b-5p, mmu-miR-466o-5p
mmu-miR-451a
mmu-miR-6391
mmu-miR-7238-5p
mmu-miR-7036b-5p
mmu-miR-29b-3p
mmu-let-7c-5p
mmu-miR-196b-5p
mmu-miR-6241
mmu-miR-6244
mmu-miR-8102
mmu-miR-6933-5p
mmu-miR-17-3p
mmu-miR-290b-5p
mmu-miR-181a-5p
mmu-miR-711
mmu-miR-665-3p
mmu-miR-29c-3p
mmu-miR-6941-5p
mmu-miR-5122
mmu-miR-8099
mmu-miR-141-3p
mmu-miR-30b-5p
mmu-let-7j
mmu-miR-107-3p
mmu-miR-700-3p
mmu-miR-15a-3p
mmu-miR-138-5p
mmu-miR-6908-5p
mmu-miR-106a-5p
mmu-miR-18b-5p
mmu-miR-196a-5p
mmu-miR-6898-5p
mmu-miR-744-5p
mmu-miR-6975-5p
mmu-miR-7650-5p
mmu-miR-320-3p
mmu-miR-181d-5p
mmu-miR-6348
mmu-let-7d-5p
mmu-miR-6982-5p
mmu-miR-8119
mmu-miR-2861
mmu-miR-3535
mmu-miR-210-3p
mmu-miR-92a-3p
mmu-miR-7026-5p
mmu-miR-17-5p
mmu-miR-6925-5p
mmu-miR-5130
mmu-miR-466a-5p, mmu-miR-466p-5p
mmu-miR-25-5p
mmu-miR-30e-5p
mmu-miR-7015-5p
mmu-miR-30d-5p
mmu-miR-764-3p
mmu-miR-466k
mmu-miR-375-5p
mmu-miR-466n-5p
mmu-miR-204-3p
mmu-let-7i-5p
mmu-miR-6537-3p
mmu-miR-29b-2-5p
mmu-miR-1957b
mmu-miR-7677-5p
mmu-miR-6912-5p
mmu-miR-23a-3p
mmu-miR-361-5p
mmu-miR-3968
mmu-miR-92b-3p
mmu-miR-1983
mmu-miR-3963
mmu-miR-30c-1-3p
mmu-miR-208a-5p
mmu-miR-92b-5p
mmu-miR-5126
mmu-miR-692
mmu-miR-195a-3p
mmu-miR-34c-3p
mmu-miR-3472
mmu-miR-7117-3p
mmu-miR-3105-5p
mmu-miR-300-3p
mmu-miR-466q
mmu-miR-5099
mmu-miR-7082-3p
mmu-miR-3068-5p
mmu-miR-3470b
mmu-miR-3473a
mmu-miR-674-3p
mmu-miR-6904-5p
mmu-miR-344f-3p
mmu-miR-5121
mmu-miR-7227-3p
mmu-miR-6238
mmu-miR-3471
mmu-miR-3470a
mmu-miR-7667-5p
mmu-miR-1247-3p
mmu-miR-7b-3p
mmu-miR-3108-3p
mmu-miR-466o-3p
mmu-miR-330-3p
mmu-miR-92a-2-5p
mmu-miR-7212-3p
mmu-miR-466b-3p,mmu-miR-466c-3p,mmu-miR-466p-3p
mmu-miR-1894-5p
mmu-miR-376b-3p
mmu-miR-5621-5p
mmu-miR-3547-3p
mmu-miR-5118
mmu-miR-485-3p
mmu-miR-7228-5p
mmu-miR-7663-5p
mmu-miR-1195
mmu-miR-195b
mmu-miR-7667-3p
mmu-miR-764-5p
mmu-miR-181b-1-3p
mmu-miR-147-5p
mmu-miR-3060-3p
mmu-miR-214-3p
mmu-miR-3473e
mmu-miR-7234-3p
mmu-miR-488-5p
mmu-miR-665-5p
mmu-miR-6976-3p
mmu-miR-1191b-3p
mmu-miR-7082-5p
mmu-miR-125b-1-3p
mmu-miR-6996-5p
mmu-miR-7671-5p
mmu-miR-5620-3p
mmu-miR-706
mmu-miR-7225-3p
mmu-miR-669a-3p, mmu-miR-669o-3p
mmu-miR-1192
mmu-miR-3964
mmu-miR-146a-5p
mmu-miR-6380
mmu-miR-6355
mmu-miR-3475-5p
mmu-miR-1224-3p
mmu-miR-7074-3p
mmu-miR-7020-3p
mmu-miR-6346
mmu-miR-6540-5p
mmu-miR-7648-5p
mmu-miR-6546-3p
mmu-miR-6958-3p
mmu-miR-434-5p
mmu-miR-5124b
mmu-miR-6406
mmu-miR-669d-3p
mmu-miR-5128
mmu-miR-7225-5p
mmu-miR-3473b

Figure S1. Heatmap represented the expression values of miRNAs that were differentially expressed in LTL high dose group (200 mg/kg) compared with the control group by microarray analysis of H460 tumor xenografts.
